# Eukaryotic Richness in the Abyss: Insights from Pyrotag Sequencing

**DOI:** 10.1371/journal.pone.0018169

**Published:** 2011-04-04

**Authors:** Jan Pawlowski, Richard Christen, Béatrice Lecroq, Dipankar Bachar, Hamid Reza Shahbazkia, Linda Amaral-Zettler, Laure Guillou

**Affiliations:** 1 Department of Genetics and Evolution, University of Geneva, Geneva, Switzerland; 2 Laboratoire de Biologie Virtuelle, Université de Nice, UMR 6543, Nice, France; 3 Japan Agency for Marine-Earth Science and Technology, Yokosuka, Japan; 4 Departamento de Engenharia Electronica e Informatica, Universidade do Algarve, Faro, Portugal; 5 Marine Biological Laboratory, The Josephine Bay Paul Center for Comparative Molecular Biology and Evolution, Woods Hole, Massachusetts, United States of America; 6 Station Biologique de Roscoff, UMR 7144, Roscoff, France; Argonne National Laboratory, United States of America

## Abstract

**Background:**

The deep sea floor is considered one of the most diverse ecosystems on Earth. Recent environmental DNA surveys based on clone libraries of rRNA genes confirm this observation and reveal a high diversity of eukaryotes present in deep-sea sediment samples. However, environmental clone-library surveys yield only a modest number of sequences with which to evaluate the diversity of abyssal eukaryotes.

**Methodology/Principal Findings:**

Here, we examined the richness of eukaryotic DNA in deep Arctic and Southern Ocean samples using massively parallel sequencing of the 18S ribosomal RNA (rRNA) V9 hypervariable region. In very small volumes of sediments, ranging from 0.35 to 0.7 g, we recovered up to 7,499 unique sequences per sample. By clustering sequences having up to 3 differences, we observed from 942 to 1756 Operational Taxonomic Units (OTUs) per sample. Taxonomic analyses of these OTUs showed that DNA of all major groups of eukaryotes is represented at the deep-sea floor. The dinoflagellates, cercozoans, ciliates, and euglenozoans predominate, contributing to 17%, 16%, 10%, and 8% of all assigned OTUs, respectively. Interestingly, many sequences represent photosynthetic taxa or are similar to those reported from the environmental surveys of surface waters. Moreover, each sample contained from 31 to 71 different metazoan OTUs despite the small sample volume collected. This indicates that a significant faction of the eukaryotic DNA sequences likely do not belong to living organisms, but represent either free, extracellular DNA or remains and resting stages of planktonic species.

**Conclusions/Significance:**

In view of our study, the deep-sea floor appears as a global DNA repository, which preserves genetic information about organisms living in the sediment, as well as in the water column above it. This information can be used for future monitoring of past and present environmental changes.

## Introduction

The development of massively parallel sequencing (pyrotag sequencing) has opened new avenues for exploring microbial and meiofaunal diversity in time and space [1]–[4]. Several studies used pyrosequencing to assess the diversity of bacteria and archaea in the marine environment [2], [5]. Yet only few of them included eukaryotic sequences [6] or focused exclusively on their diversity [3], [7]. Until now, no one has applied pyrosequencing to examine eukaryotic diversity at the abyssal sea floor.

The deep-sea benthic environment is one of the most diverse and extensive habitats on Earth. Many deep-sea taxa are extremely speciose, but their distribution is patchy and their abundance is usually not very high [8]. Some deep-sea species seem to have very large geographic ranges [9]. However, existing molecular biogeographic data are sparse. Environmental DNA surveys of the deep-sea floor have revealed high richness of deep-sea micro-eukaryotes. These studies focused on extreme environments, including hydrothermal vents [10]–[13], cold methane seeps [14], or hypersaline anoxic basins [15]–[16]. Some studies examined select groups of deep-sea protists, such as diplonemids [17] or ciliates [18]. Very little is known about the deep-sea benthic eukaryotic communities in polar regions [19]–[20] and abyssal plains [21]. Moreover, all these studies analysed clone libraries with a limited number of sequence data available.

As part of the International Census of Marine Microbes (ICoMM:http://icomm.mbl.edu) community sequencing project, we examined eukaryotic 18S rRNA gene richness in six deep-sea stations in the Arctic and Southern Oceans (Table 1). We obtained 108,632 18S rRNA gene V9-hypervariable region sequence reads. We clustered the reads into 8,309 OTUs, which spanned the breadth of the eukaryotic tree of life, including many sequences, which originated from photosynthetic taxa. We discuss the efficiency of V9 sequences for identification of eukaryotes and we argue that the DNA preserved in the deep-sea sediments reveals not only the diversity of benthic fauna but also that of organisms deposited on the deep-sea floor from the surface waters.

**Table 1:** Geographic coordinates of sampling sites and collecting dates.

Sample	Locality	Latitude	Longitude	Depth	Date
DSE1	Weddell Sea	65°19′90 S	48°05′55 W	4060 m	09/03/2002
DSE2	Weddell Sea	58°24′96 S	25°00′94 W	2292 m	22/03/2002
DSE3	Weddell Sea	58°50′81 S	23°58′55 W	6326 m	24/03/2002
DSE4	Arctic Ocean	83°06′73 N	86°17′87 E	3148 m	22/08/2007
DSE5	Arctic Ocean	84°09′62 N	60°53′42 E	3700 m	12/08/2007
DSE6	Arctic Ocean	82°06′19 N	69°03′62 E	686 m	17/08/2007

## Results

### Sequence data

We obtained 124,671 reads for all samples (Table 2). About 13% of these reads were assigned to Archaea, Bacteria or eukaryotic genes other than rRNA and were discarded from analyses. This has been acknowledged in previous studies to be the result of primers designed to capture the largest eukaryotic diversity possible [3]. The total number of eukaryotic reads was 108,632, ranging from 10,659 in sample DSE4 to 30,608 in DSE1. After strict dereplication, this number was reduced to 29,627 unique sequences, ranging from 2,769 in DSE4 to 7,499 in DSE3. By clustering the unique sequences differing by 3 or less nucleotides, we further reduced the number of sequences to 8,309 OTUs. The number of OTUs per sample ranged from 942 OTUs in DSE4 to 1,756 OTUs in DSE6, with a mean value of 1,385. About 70% of OTUs could be assigned to a taxonomic group, following the assignment criteria described in the methods. The highest proportion of unassigned OTUs occurred in DSE2 (31%), with the values ranging from 19 to 29% for other samples.

**Table 2:** Number of reads, tags and OTUs.

	DSE1	DSE2	DSE3	DSE4	DSE5	DSE6	Total
Total 454 reads	35280	15847	33731	12973	12777	14063	124671
archaeal reads	519	408	1836	932	347	292	4334
bacterial reads	4117	1466	2950	1256	700	903	11392
non-rRNA reads	36	81	39	126	24	7	313
eukaryotic reads	30608	13892	28906	10659	11706	12861	108632
total unique tags	6956	4293	7499	2769	3809	4301	29627
total OTUs (k = 3)	1635	1542	1224	942	1210	1756	8309
total assigned (>80%)	1157	1065	992	675	901	1255	6045
unassigned (<80%)	478	477	232	267	309	501	2264

### Taxonomic richness

**Figure 1:**
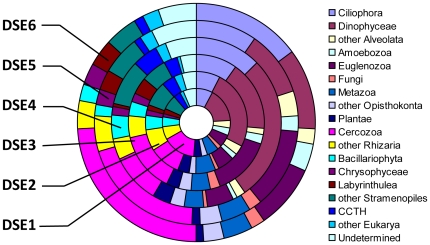
The abundance of major groups of eukaryotes in abyssal Arctic and Southern Ocean environmental DNA.

Table 3 and Figure 1 contain the distribution of assigned OTUs among the major taxonomic groups of eukaryotes. The distinction of major groups followed the commonly accepted higher-level classification of eukaryotes [22] modified according to a phylogenomic study [23]. We subdivided some large assemblages to better illustrate the proportion of common groups, for example Ciliophora and Dinophyceae in the case of Alveolata, Fungi and Metazoa in the case of Opisthokonta, Cercozoa in the case of Rhizaria, and Bacillariophyta, Chrysophyceae, and Labyrinthulea in the case of Stramenopiles. The other taxa belonging to larger assemblages were combined into separate groups. For example, we included Foraminifera and Radiolaria in “other Rhizaria”, Choanoflagellata and Ichtyosporea in “other Opisthokonta”, and Pelagophyceae, Dictyochophyceae, Bolidophyceae and others in “other Stramenopiles”. A new group CCTH [23], called also Hacrobia [24] included OTUs assigned to Cryptophyta, Haptophyta, Telonemia and Centroheliozoa. We placed a few eukaryotic groups (Apusozoa, Katablepharids and Picobiliphyta), whose position is not established yet, in “other Eukarya”. This group comprises a few OTUs assigned to Heterolobosea that are usually grouped with Euglenozoa in the supergroup of Excavata. We placed OTUs with conflicting taxonomic assignments in an “undetermined” group.

**Table 3:** Taxonomic assignment of OTUs to major eukaryotic groups.

	DSE1	DSE2	DSE3	DSE4	DSE5	DSE6
Ciliophora	172	103	125	66	68	98
Dinophyceae	127	110	275	112	226	203
other Alveolata	24	42	17	10	27	45
Amoebozoa	43	26	8	15	16	45
Euglenozoa	101	136	50	96	48	75
Fungi	22	22	7	16	10	24
Metazoa	46	50	37	31	57	71
other Opisthokonta	32	37	27	20	19	35
Plantae	12	14	34	20	33	47
Cercozoa	279	210	94	96	90	196
other Rhizaria	44	60	39	35	61	77
Bacillariophyta	26	19	45	23	41	59
Chrysophyceae	34	26	16	14	16	19
Labyrinthulea	42	34	6	27	15	31
other Stramenopiles	52	65	85	41	71	88
CCTH^1^	15	12	48	22	38	41
other Eukarya^2^	26	22	20	5	23	22
Undetermined	60	77	59	26	42	79
Subtotal assigned	1157	1065	992	675	901	1255
Unassigned	478	477	232	267	309	501
Total	1635	1542	1224	942	1210	1756

Notes:

1 CCTH group comprises Cryptophytes, Centroheliozoa, Telonemida and Haptophyta as defined by Burki, et al. (2009).

2 Other Eukarya comprises Apusozoa, Picobiliphyta, Katablepharismids, Heterolobosea.

All major taxonomic groups of eukaryotes were present in our samples (Figure 1). Four groups: Dinophyceae, Cercozoa, Ciliophora and Euglenozoa dominated the assemblage, accounting together for 51% of total assigned OTUs. The relative frequencies of these groups varied between samples. Cercozoan assigned OTUs dominated in DSE1 (24%), DSE2 (20%), while Dinophyceae dominated in DSE3 (28%), DSE4 (17%), and DSE5 (25%). Both groups formed 16% of total assigned OTUs in DSE6. The proportion of Ciliophora varied from 8% (DSE5, DSE6) to 15% (DSE1), while that of Euglenozoa reached 14% in DSE4 and 13% in DSE2, but ranged from 5 to 9% in other samples. Other common groups were Metazoa (5–6%), Bacillariophyta (2–5%), other Stramenopiles (4–9%) and Foraminifera + Radiolaria (other Rhizaria) (4–7%). All other groups did not exceed 5% in any of the samples, with particularly low abundance of Fungi (<2%) and Amoebozoa (<4%).

**Figure 2:**
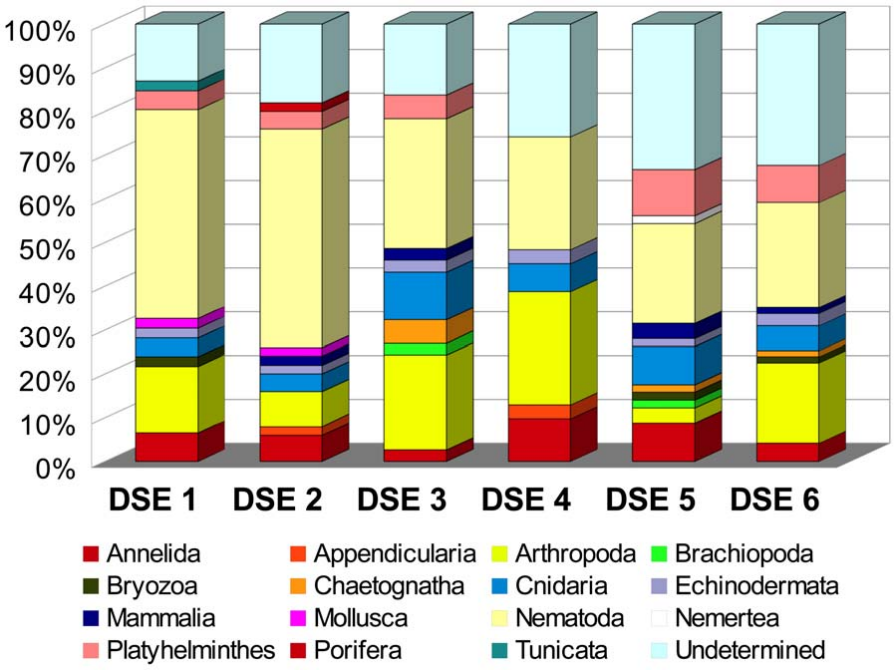
Taxonomic distribution of OTUs assigned to Metazoa.

We explored the taxonomic distribution of Metazoa in greater detail (Figure 2, Table S1). The number of metazoan OTUs ranged from 31 (DSE4) to 71 (DSE6). They could be assigned to 15 different phyla, but seven were represented by not more than three OTUs. In the case of Nemertea, Porifera and Tunicata, only a single OTU was found. By far the most abundant were Nematodes, which formed up to 50% of all metazoan OTUs (DSE2). We also found several OTUs of Annelida, Arthropoda (mainly Copepoda), Cnidaria and Platyhelminthes. Interestingly, the number of undetermined metazoans was relatively low in DSE1-4, but reached almost 30% in DSE5 and DSE6.

**Figure 3:**
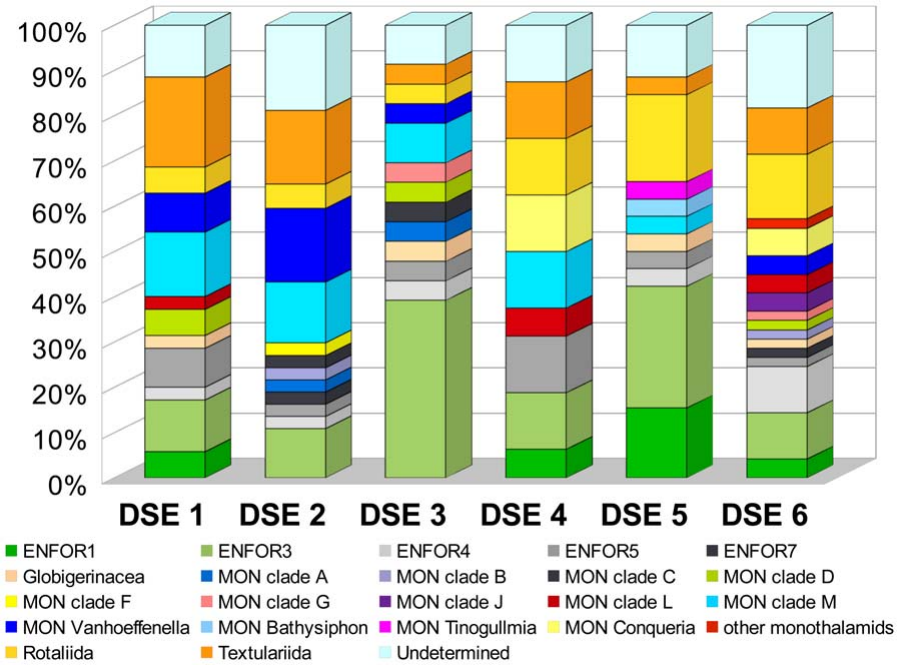
Taxonomic distribution of OTUs assigned to Foraminifera.

We were also able to assign greater taxonomic resolution to our foraminiferal OTUs, using an in-house database of foraminiferal SSU rRNA gene sequences in the Pawlowski laboratory. Foraminiferal OTUs were not very abundant, ranging in number from 16 to 49 (Table S2). However, their identification at a finer level was quite reliable compared to other groups, the proportion of undetermined OTUs varied between 11% and 19% (Figure 3). We distinguished 5 clades of environmental sequences (ENFOR), 9 clades of monothalamous (single-chambered) species (MON) and 4 monothalamous genera (MON), following Pawlowski et al. [25]. The OTUs assigned to the multi-chambered species were placed in one of the 3 groups: planktonic Globigerinaceae, benthic calcareous Rotaliida, and benthic agglutinated Textulariida. The most abundant were the OTUs assigned to environmental clades (ENFOR) and to monothalamids (MON). These two categories accounted for almost 80% in some samples (DSE3). The multi-chambered rotaliids and textulariids, accounted for 14 and 20%, respectively.

### Identification of planktonic OTUs

In order to examine the origin of eukaryotic richness, we estimated the proportion of environmental sequences corresponding to the organisms that are not known to inhabit the deep-sea floor. At first, we selected the taxonomic groups that are known phototrophs. We included three groups of Plantae (Chlorophyta, Rhodophyta and Glaucophyta), as well as the haptophytes, the picobiliphytes, and various stramenopiles (Bacillariophyta, Pelagophyceae, Dictyochophyceae, Bolidophyceae, Phaeothamniophyceae, Pinguiophyceae, Raphidophyceae, Phaeophyceae) that carry out photosynthesis. Our selection comprised also some phototrophic genera of Dinophyceae and Radiolaria that usually live in symbiosis with microalgae and are considered as having an exclusively planktonic mode of life.

In total, 710 OTUs were assigned to the phototrophic taxa and radiolarians (Table 4). Their numbers varied from 68 to 168 per sample. The most abundant were the OTUs of diatoms (Bacillariophyta) and plants (mainly Chlorophyta). In some samples we also found many radiolarians, haptophytes and picobiliphytes. On the other hand, the phototrophic stramenopiles other than diatoms were rare. There were few dinoflagellates that could be reliably assigned to photosynthetic genera, but this was due mainly to the difficulties in assigning dinoflagellate sequences to a finer taxonomic level.

**Table 4:** Number of OTUs assigned to planktonic taxa.

Planktonic taxa	DSE1	DSE2	DSE3	DSE4	DSE5	DSE6
Dinophyceae	5	5	30	5	14	10
Plantae	12	14	34	20	33	47
Haptophyta	4	2	17	10	14	11
Picobiliphyta	7	8	16	6	18	10
Radiolaria	8	15	15	12	24	18
Bacillariophyta	26	19	45	23	41	59
Bolidophyceae	0	3	1	0	1	0
Dictyochophyceae	1	4	3	0	2	4
Pelagophyceae	1	0	3	0	3	2
Phaeophyceae	3	2	2	2	4	4
Phaeothamniophyceae	1	2	0	0	1	0
Pinguiophyceae	0	0	0	0	0	1
Raphidophyceae	0	0	2	0	0	1
Total phototrophic taxa	68	74	168	78	155	167
**Environmental OTUs:**						
Marine plankton*	274	228	395	174	288	272
Marine sediment only	147	134	75	92	64	105
Freshwater & soil	44	37	29	21	27	37
Putative planktonic OTUs	342	302	563	252	443	439
Percentage of all assigned OTUs	**30%**	**28%**	**57%**	**37%**	**49%**	**35%**

*including the OTUs found in the sediment, but without the planktonic taxa listed above.

**Figure 4:**
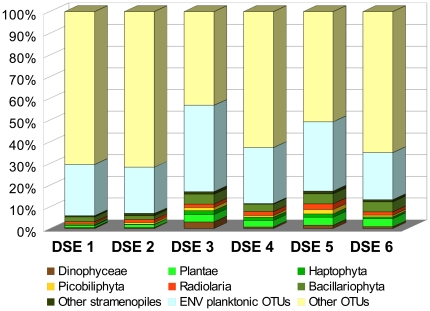
The abundance of OTUs assigned to phototrophic taxa and planktonic environmental sequences.

In addition to identifying the phototrophic taxa, we also searched for the sequences that were similar to the environmental sequences obtained in other studies of marine plankton. An OTU was considered of planktonic origin if it was >90% similar to the sequences found previously in any clone libraries from surface and water column samples. The number of these putative planktonic OTUs ranged from 220 (DSE4) to 511 (DSE3). After removing the OTUs belonging to the phototrophic taxa listed above, the number of planktonic OTUs averaged 272, reaching up to 395 OTUs in DSE3 sample (Table 4). It should be noted that the samples having the highest number of planktonic OTUs identified in comparison with other environmental studies were also those, in which the phototrophic taxa were the most abundant. When we added the OTUs assigned to photosynthetic taxa and those found in plankton samples, we observed that their proportion exceeded 30% in all but one sample (Figure 4). The highest proportion was observed in sample DSE3, in which the putative planktonic OTUs reached 57% of the total number of assigned OTUs.

### Testing widespread distribution of eukaryotic OTUs

**Figure 5:**
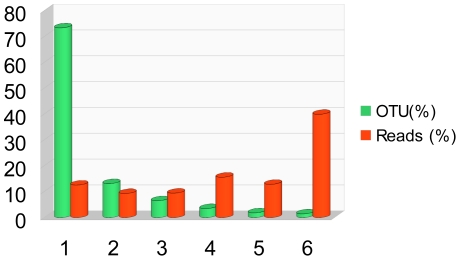
The abundance of OTUs and reads in 1 to 6 samples.

In order to test how widely distributed the OTUs identified in our study were, we compared the samples from the Southern (DSE 1–3) and Arctic (DSE 4–6) Oceans. Our analyses showed that only 84 OTUs (1.4%) occurred in both regions i.e. present in all six samples (Table 5). The majority of OTUs (73%) were present in one sample only, and only 7% were present in more than 3 samples. Interestingly, the widely distributed OTUs were represented by higher numbers of reads. In particular, the OTUs occurring in both poles totaled 40% (43,467) of reads (Fig. 5). The proportion of reads was even higher (78%) if the OTUs present in a minimum of one sample of each region were considered (Table 5). On the other hand, the “endemic” OTUs present exclusively in one region were rare. We found only 37 and 45 OTUs present in all three samples of the Arctic and Southern Ocean samples, respectively. The number of reads corresponding to these OTUs was relatively small (3,897).

**Table 5:** Number of OTUs and reads found in 1 to 6 samples, as well as in all samples or minimum 1 sample of both regions (ARC + ANT), or exclusively one region (ARC - Arctic, ANT - Antarctic).

Samples	OTUs	Reads
6	84	43467
5	108	14013
4	204	17006
3	381	10292
2	767	10157
1	4275	13697
**Total**	**5819**	**108632**
All samples:		
ARC+ANT	84	43467
ARC only	37	1143
ANT only	45	2754
Min 1 sample:		
ARC+ANT	1071	85235
ARC only	2051	8489
ANT only	2697	14908

In order to identify widespread and “endemic” OTUs we carefully checked their assignment at the species level. Surprisingly, we found that the majority of widespread OTUs (76%) could be assigned to planktonic taxa, usually at a very high level of similarity (>95%). Almost all taxonomic groups were represented, with most of “polar” OTUs assigned to Dinophyceae, Cercozoa and the CCTH group (Table 6). On the contrary, the taxonomic assignment of “endemic” OTUs was much less precise (rarely exceeding 90%). The proportion of OTUs that could not be reliably (similarity <80%) assigned to any particular group reached up to 53% in Southern Ocean samples. Among the few assigned OTUs, we found mainly the parasitic groups, such as Haplosporidia, and the uncultured eukaryotes reported from the other deep-sea sediments samples, while planktonic taxa were rare, especially in Southern Ocean samples.

**Table 6:** Taxonomic identification of “bipolar” and “endemic” OTUs, present in all 6 samples (ANT + ARC) or 3 samples of one region exclusively (ANT only, ARC only).

	ANT + ARC	ANT only	ARC only
Ciliophora	3	4	0
Dinophyceae	17	3	5
other Alveolata	2	0	0
Amoebozoa	2	0	1
Euglenozoa	3	4	1
Fungi	0	0	0
Metazoa	2	0	3
other Opisthokonta	1	0	0
Plantae	4	0	0
Cercozoa	8	4	3
other Rhizaria	3	0	2
Bacillariophyta	4	0	1
Chrysophyceae	1	1	0
Labyrinthulea	0	0	1
other Stramenopiles	6	2	0
CCTH	9	0	3
other Eukarya	0	1	0
Undetermined	8	2	0
Unassigned	11	24	17
Total	84	45	37
Planktonic (%)	76	9	22
Unassigned (%)	13	53	46

Percent of planktonic and unassigned OTUs is indicated.

## Discussion

### Advantages and pitfalls of the V9 domain

The choice of the 18S hypervariable region for pyrosequencing is extremely important and an ongoing topic for discussion [3], [26]. Environmental studies usually target either the V4 hypervariable region, which is characterized by particularly rapid rates of evolution and is subject to extreme variation in length [27] or the V9 region, which is much shorter and shows less length heterogeneity [3], [6], [7]. The latter studies showed that V9 is a relatively good compromise between the large range of eukaryotic diversity retrieved with this domain and the level of taxonomic identification. Our study confirms this view. The universal primers used for amplification of V9 recognize practically all eukaryotic phyla, even those that are well known to be particularly difficult to amplify, like Amoebozoa or Foraminifera [3]. Their recognition spectrum is much larger than that of primers commonly used for amplification of the V4 domain, which miss, among others,the excavates and foraminiferans. Although the V9 primers used here amplify also some Bacteria and Archaea, the number of their reads is not high (Table 2) and they can be identified *in silico* and discarded.

The comparison between V4 and V9 regions shows much higher diversity level obtained by analysis of V4 compared to V9 [26]. This could suggest that V4 is more variable than V9 in some taxonomic groups. Indeed, our data contain several examples of species having identical sequences in V9 region (for example in the genus *Phaeocystis*). Even the eukaryotes with rapidly evolving rRNA genes, such as benthic Foraminifera, comprise species that cannot be distinguished in the V9 domain [28]. However, the higher diversity of V4 may also be due to other factors. As demonstrated by Stoeck et al. [26] in the case of dinoflagellates, the primers used for amplification of both regions may detect very different taxonomic profiles, what may strongly influence the number of different OTUs. Moreover, the higher diversity observed in analyses of V4 region may be related to technical errors caused by higher number of homopolymers in longer and structurally more complex V4 region [26].

The short length of the V9 region and lack of specific signatures for higher-level taxa may cause a certain number of conflicts in taxonomic assignments, especially when there is no good match for a given sequence in the reference database. Some groups, such as Ameobozoa, are particularly difficult to recognize. There are also conflicts between the sequences of some distinct taxonomic groups. For example, our sequence DSE2-4618 is 99% identical to the diatom *Stellarima microtrias* (EU090011) and 98% to the sequence of a bivalve *Thracia meridionalis* (AY192700). However, such conflicts are rare and often due to the misclassification of a sequence in GenBank due to the chimeric character of one of the sequences (*T. meridionalis* in the example cited above). Finally, some taxa might be entirely missing from the reference database, because the V9 domain was not sequenced for these groups. We attribute the high number of unassigned OTUs, ranging from 18 to 30% in our samples to the lack of a proper reference sequence present in public databases.

A final issue which needs to be raised are errors generated during pyrosequencing. The 454 sequencing method does not call bases directly but nucleotide flows are indicated by a light signal. For each flow representing a homopolymer the brightness of the light is proportional to the length of the homopolymer. The brightness of the light is easy to mis-calibrate, especially for long homopolymers. A method has been proposed to correct this problem [29]. Apart from the very high computing power necessary, PyroNoise was designed to analyse sequences for which the distal primer was not reached. As a result, it trims the various sequences at an approximately equal length, reducing the length of the longer sequences; this can therefore be problematic when taxonomy is assigned using a minimal percent of similarity with the reference sequences. In our case, we required the presence of exact matches to primer sequences at both the proximal and the distal ends of our amplicons as an indication that the sequencing was good. Consequently, both the 5′ and the 3′ ends of the sequences are truly orthologous in all sequences and trimming the sequences would have lead to a heavy loss of information. Instead we devised a new method in which the distance between two sequences was calculated using pair-wise global alignments (Needleman*-*Wunsch) in which differences in length for homopolymers were not counted as differences.

### Hidden diversity of eukaryotes

Our high-throughput sequencing study confirms that microbial eukaryotic community diversity in deep-sea sediments is extremely rich. Even if the taxonomic resolution of V9 is limited at the genus level, the number of observed eukaryotic OTUs recovered in our study was impressive. Almost all samples except DSE4 yielded more than 1000 distinct OTUs. Even when sequences were clustered at up to 8 differences and clusters with a single sequence removed (as they may represent sequencing errors), more than 400–500 OTUs were observed in every sample. Compared to other studies [7], the proportion of metazoans was relatively limited (<71 OTUs). The majority of OTUs belonged to Alveolata and Euglenozoa in agreement with previous studies based on environmental cloning and sequencing [12]. We found almost all taxa that were previously reported from the deep-sea bottom environment [11], [14]. Compared to these studies, however, the proportion of unassigned and undetermined sequences in our data was much higher. Although it is difficult to phylogenetically analyze these very short V9 sequences, many of our OTUs have been assigned to the lineages that are known exclusively from environmental sequences, suggesting that cryptic diversity may be an important component in our data.

Among the groups with the highest number of OTUs in our samples, cryptic diversity was particularly important in Cercozoa. This poorly known group consists of an assemblage of heterotrophic flagellate and amoeboid protists [30]. Its diversity seems enormous as documented by numerous new species recently described from laboratory cultures [31] and many new lineages revealed by environmental studies [32]. In view of these studies it is not surprising to find the Cercozoa dominating some of our assemblages (DSE1, DSE2). It is more difficult to identify the cercozoan species present in our samples. Many of them belong to the novel lineages Endo-2 and Endo-3, which branch close to Haplosporidia [31]. However, a large proportion of OTUs assigned to Cercozoa remained unidentified at a finer level of taxonomic resolution.

Another taxonomic group that shows high cryptic diversity are Foraminifera. Compared to the Cercozoa, the deep-sea foraminifera have been studied for more than a hundred years and many species have been described from deep-sea sediment samples. Therefore, it was quite surprising to find that the majority of foraminiferal OTUs in our material did not belong to well-established taxonomic groups. These groups included mainly multi-chambered calcareous Rotaliida and agglutinated Textulariida, whose tests are well preserved in sediment samples. The proportion of rotaliids and textulariids in our samples averaged 20%. On the other hand, the vast majority of foraminiferal OTUs belonged to the non-identified groups of monothalamous (single-chambered) taxa or to the environmental clades (ENFOR). The ENFOR clades are composed almost exclusively of sequences found in environmental studies [19], [33]. The morphology and biology of these organisms is unknown. They are probably tiny, having no theca or organic one and thus poorly preserved in the sediment samples or during sampling. Some recent studies showed an abundance of small-sized organic-walled allogromiids at the deep-sea bottom [34]. Most of our environmental sequences probably belonged to this group.

### Origins of eukaryotic DNA in the deep-sea sediments

Taxonomic analysis of the eukaryotic diversity found in our samples suggested that many OTUs do not belong to the organisms endemic to the deep-sea bottom. Among them were many phototrophs that dwell in the surface waters and sink to the bottom, where their DNA is preserved. Other authors [11] reported the presence of the phototrophic taxa in clonal environmental studies of deep-sea sediment, but their importance was not evaluated until now. Although some authors considered them to be of minor importance in deep-sea diversity estimation [21], our study shows that the phototrophs (including the planktonic species that bear photosynthetic symbionts, such as radiolarians) can form up to 17% of the total number of assigned OTUs. The proportion of DNA originating from the plankton was even higher if we add the OTUs that show high similarity (>90%) to taxa that have been found in environmental plankton sampling. In total, more than 30% of OTUs could have planktonic origins and this value is probably an underestimation.

Planktonic taxa were particularly abundant (76%) among the OTUs present in all 6 samples (Table 6). Some of these OTUs could be assigned to well known pan-oceanic phototrophic and heterotrophic taxa, such as clade A and D of *Micromonas pusilla* [35], *Thallasiosira*, *Phaeocystis*, *Aureococcus, the ciliate Strombidium, the cercozoan Cryothecomonas, marine stramenopiles* MAST 1A, 1C and 9A, and MALV I and II. Others may represent polar endemic species [36] such as for example, the DSE1-7905 that has 100% identity with the Arctic *Chaetoceros neogracile* ArM004 [37]. However, the V9 region is not variable enough to ensure that these OTUs do not represent cryptic species or different populations of the same species and that their presence at both poles is in fact an artifact of using slowly evolving 18S rRNA gene. Remarkably, these planktonic OTUs are not very numerous (1.4%), yet they contribute almost 40% of total number of reads. Their great abundance in the water seems reflected by large amounts of their DNA deposited in the sediment.

In addition to the DNA of planktonic organisms, many OTUs identified in our study probably correspond to benthic organisms, whose DNA was preserved in the deep-sea sediments. For example, the large diversity of metazoans found in our samples contrasts with a very small size of sediment samples (0.35 or 0.7 g), from which DNA was extracted. We cannot exclude the possibility that some of these sequences, especially the mammalian ones, were the result of laboratory contamination. Some others originate possibly from planktonic groups (Appendicularia, Chaetognatha, which have only one benthic genus, as well as some Arthropoda, Cnidaria or Mollusca). However, the majority of metazoan OTUs correspond to the typical benthic fauna, including nematodes, brachiopods, bryozoans, poriferans and echinoderms. Most likely, many of these OTUs were obtained from the trace DNA present in tissue fragments, mucus, fecal pellets and other metazoan remnants or from the extracellular DNA, considered a major source of DNA at the deep-sea bottom [38].

Extracellular DNA and DNA from resting stages and cysts could also explain the high diversity of other groups of eukaryotes. However, this does not mean that there are no autochthonous eukaryotic fauna living at the deep-sea bottom. The diversity of some deep-sea protists, for example benthic foraminifera, is well documented [39]. There are also few reports of deep-sea flagellates [40], ciliates [18] and amoebae (Kudryavtsev, pers. comm.). Some taxonomic groups of Euglenozoa and Ciliophora are considered endemic to the deep-sea environment [12], [21]. This is confirmed by rareness of their sequences in water samples from the surface and greater depths [6]. This is also in agreement with the abundance of both groups in our samples, where they form up to 14% and 15% of total assigned OTUs, respectively. However, little is known about the ecology of the deep-sea representatives of these groups. Some euglenozoan genera are known to be parasites and their abundance in sediment samples could be due to the massive release of spores from infected and dead hosts. This may also explain the abundance of parasitic taxa, such as *Amoebophrya* and Syndiniales (Dinoflagellates), or haplosporidians (Cercozoa) in our samples, as well as in previous studies [41].

The diversity of autochtonous deep-sea species is still largely unknown. This is particularly true for abyssal plains that have been much less sampled than the hydrothermal vents or other extreme deep-sea habitats. If we exclude the putative planktonic taxa, there are still about 4,000 OTUs that possibly correspond to deep-sea benthic species. Many of them were assigned at a low level (<90%) of taxonomic certainty or remained unassigned (<80%) showing the paucity of the available database. Compared to planktonic OTUs, the number of reads corresponding to benthic OTUs is much lower and their distribution seems much more restricted. The deep-sea benthic OTUs may be globally distributed but their abundance is too low to be detected in every sample. For example, the widespread benthic foraminiferal species *Epistominella exigua* [42] was found in all Southern Ocean samples (DSE 1–3) but not in the Arctic Ocean, despite that being reported there, albeit not in the same sampling sites [9]. The number of samples analysed here is too small to make conclusions about the distribution patterns of detected OTUs.

Because of difficulties in direct observation of life at the ocean bottom and the complex interactions between the benthic and pelagic realms, the interpretation of DNA sequences recovered from deep-sea sediments is quite problematic. Clearly, the analysis of deep-sea RNA will be necessary to identify metabolically active organisms. Nevertheless, analyses of deep-sea environmental DNA are of particular interest. The DNA concentration in deep-sea sediment can be extremely high [38] and its capacity to absorb dissolved DNA is probably as good as that of a sandy beach [43]. As shown by this and other studies, DNA deposited at the deep-sea floor represents all forms of eukaryotes living at different depths from the surface to the bottom. Therefore, its analysis provides unique insight into the richness of marine life, including both benthic and pelagic domains. Moreover, as it has been shown that DNA can be preserved in marine sediment over time [44], [45], the environmental study of ancient deep-sea DNA samples will provide a new tool to explore the past and present history of marine life.

## Materials and Methods

### Sampling, DNA extraction, PCR amplification and 454 sequencing

Samples were collected from the Arctic and Southern Oceans, at depths ranging from 686 to 6326 m (Table 1). Sediment was taken from the upper layer (1–2 cm depth) of the multicore samples and frozen immediately after collection at −20°C. The samples were transferred frozen to the laboratory in Geneva and stored at −80°C. Small subsamples, of 0.35 or 0.7 g, were extracted for DNA using a Power Soil DNA Isolation Kit (MO BIO Laboratories, Carlsbad, CA). PCR amplification and pyrosequencing followed the protocol of [3]. Tag sequences have been deposited in the National Center for Biotechnology Information (NCBI) Sequence Read Archive (SRA) under the accession number SRP001212 [46]. The environmental data and information about marker gene are presented in MIMARKS compliant table (Table S3).

### Sequence data processing

#### Dereplication and clustering

Within each sample, sequences were first strictly dereplicated; i.e. exactly identical sequences (occurrences) were grouped as a single sequence (uniques) and data were sorted by decreasing abundances. Unique sequences were then dereplicated at k differences. During this process the most abundant sequence was first taken as a seed and a less abundant sequence was grouped with it if both sequences were similar at k differences or less. Then the next most abundant unique sequence was used as a seed and the remainder were compared to the new seed, until all sequences had been analyzed. To compare two sequences, we developed a new Needleman-Wunsh algorithm in which differences were counted only if they did not correspond to differences in homopolymers lengths. For example sequences ATGTGGGGTAT and ATGTGGGTAT are grouped together at 0 differences. Indeed errors in reading homopolymers are by far the most abundant errors resulting from 454 sequencing, they can represent more than 50% of errors in SSU rRNA sequences which have many homopolymers [29], [47].

After the clustering process at k differences, some clusters are composed of a single unique sequence with 1 occurrence of a singleton; we call these sequences single-singletons. Many of these single-singletons are the results of large sequencing errors. However, because we were particularly interested in rare tag sequences present in deep-sea samples, we kept the single-singletons and used sequences clustered at k = −3 for our analyses (Figure S1).

### V9 database construction and analysis of the taxonomic properties of the domain

We extracted 8,581 V9 domains from a database containing 22,450 reference eukaryotic sequences (Guillou et al. unpublished). This database consists of curated deposited sequences annotated with up-to-date taxonomy and quality controlled to remove chimeras. Each clade was then successively extracted, aligned and compared to the primers used for amplification. These sequences were aligned using Muscle [48] and visualized using Seaview [49].

In order to check for the validity of taxonomic assignments using the V9 domain only, we performed two experiments. In the first analysis, we clustered all V9 sequences using uclust (http://www.drive5.com/usearch/) with option --optimal and ranging from 99% to 85% similarity. In each cluster, taxonomic assignments of the given sequences were compared and a consensus built. For example, a given cluster can be assigned to level 1 only, when the taxonomy only agrees at the level of domain Eukarya (i.e. phylum-level assignments are contradictory). In the second analysis, we clustered all V9 sequences using our Needleman*-*Wunsch algorithm under the same conditions used to cluster the 454 sequences. Correspondences between k values and % similarity were approximate as we do not count differences in homopolymers, but the results were very similar to assignments as described above. In all cases, this showed that even at 85% similarity levels, more than 80% of the V9 sequences are unambiguously assigned at the genus or family level, and more than 90% of the sequences are assigned at the genus level at 98% similarity or more.

### Taxonomic assignment

We assigned taxonomy to each 454 sequence by conducting BLASTN searches (using parameters -W 7 -m 7 -r 5 -q -4 -G 8 -E 6 -b 50) of each unique sequence against our V9 reference database described above. We requested an XML output with up to 30 hits, used a word size option of 7 and applied no filer in order to obtain the highest sensitivity. Each XML file was parsed to calculate the percentage of similarity between a query seed and a hit. Because BLAST does local alignments, a true percentage is often difficult to calculate therefore, we used the following equation: sum of (identities - gaps)/length of query seed. The sum was done over every non overlapping High Scoring Pair (HSP) (see BLAST documentation); the calculated percentage is therefore much more stringent than a calculation done on the first HSP only and being the division of similarity by alignment length, which can often result in spurious high percentages when HSPs concern conserved domains only. The Silva database (http://www.arb-silva.de/) is usually used for taxonomic assignments of bacterial and archaeal sequences because Silva taxonomic assignments have been carefully reviewed by experts [50]. For eukaryotic sequences however, Silva only contains the NCBI assigned taxonomy itself that may be unreliable at times.

Only unique sequences with a best BLAST hit of at least 80% sequence similarity were assigned to a taxonomic category. The remaining sequences were labeled as “undetermined”. Despite the good resolution of the V9 domain, as shown above, it is still possible that a V9 sequence will be similar to representative sequences belonging to quite distinct clades. In order to take that possibility into account, we required that 75% of the good hits share the same taxonomy. If this was not possible at the genus level, then this was required for the family level and so on. As a result some sequences could be assigned only at the domain level. All these operations were done through a pipeline written using the Python language, except the *Needleman*-*Wunsch* program which was written in C++.

The BLAST hit having the most similar sequences was also compared and seldom yielded a discrepancy in its taxonomy and the one obtained by the method described above. These analyses were run at the successive thresholds of 70, 75, 80, 85, 80, 92, 95, 96, 97, 98, 99 and 100% similarity. This allowed different estimates to be used for different clades, as we know that within protists the SSU rRNA sequences can evolve at very different rates.

In order to identify the sequences originated from surface and water column, another BLAST search was done, with similar parameters, but on a database formatted using only the eukaryotic SSU rRNA sequences of the EMBL database described as "environmental sequences". The results were analyzed and for each hit sequence above 90%, similarity, entries were analysed for information about collection sites (marine plankton, marine benthos, freshwater, soil). For each sample, these publications allowed to identify a list of environments in which similar sequences had been found.

## Supporting Information

Figure S1 **Saturation curves with (at left) and without (at right) single-singletons.**
(TIF)

Table S1 **Taxonomic composition of OTUs assigned to Metazoa.**
(DOC)

Table S2 **Taxonomic composition of OTUs assigned to Foraminifera.**
(DOC)

Table S3 **MIMARKS data.**
(XLS)
