# Supplementary figures and images for: Eukaryotic Richness in the Abyss: Insights from Pyrotag Sequencing
Source: PLoS One. 2011 Apr 4;6(4):e18169. doi: 10.1371/journal.pone.0018169 (PMC3070721; doi:10.1371/journal.pone.0018169)

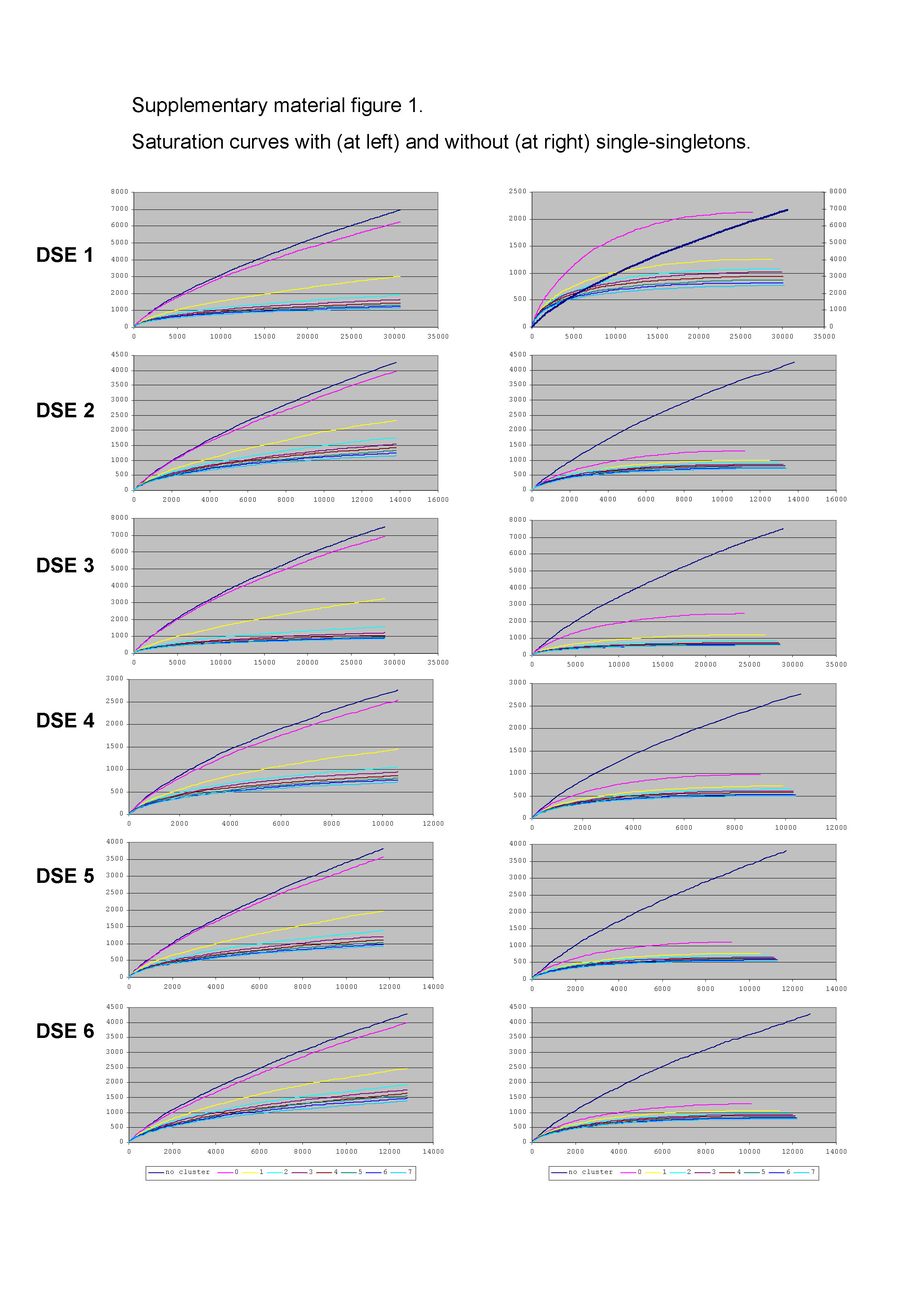

Supplement: Figure S1 — Saturation curves with (at left) and without (at right) single-singletons. (TIF) [file pone.0018169.s001.tif]
