# Supplementary material for: Eukaryotic Richness in the Abyss: Insights from Pyrotag Sequencing
Source: PLoS One. 2011 Apr 4;6(4):e18169. doi: 10.1371/journal.pone.0018169 (PMC3070721; doi:10.1371/journal.pone.0018169)
Supplement: Table S1 — Taxonomic composition of OTUs assigned to Metazoa. (DOC) [file pone.0018169.s002.doc]

Table S1.

Taxonomic composition of OTUs assigned to Metazoa

| **Metazoa** | **DSE1** | **DSE2** | **DSE3** | **DSE4** | **DSE5** | **DSE6** |
| --- | --- | --- | --- | --- | --- | --- |
| Annelida | 3 | 3 | 1 | 3 | 5 | 3 |
| Appendicularia | 0 | 1 | 0 | 1 | 0 | 0 |
| Arthropoda | 7 | 4 | 8 | 8 | 2 | 13 |
| Brachiopoda | 0 | 0 | 1 | 0 | 1 | 0 |
| Bryozoa | 1 | 0 | 0 | 0 | 1 | 1 |
| Chaetognatha | 0 | 0 | 2 | 0 | 1 | 1 |
| Cnidaria | 2 | 2 | 4 | 2 | 5 | 4 |
| Echinodermata | 1 | 1 | 1 | 1 | 1 | 2 |
| Mammalia | 0 | 1 | 1 | 0 | 2 | 1 |
| Mollusca | 1 | 1 | 0 | 0 | 0 | 0 |
| Nematoda | 22 | 25 | 11 | 8 | 13 | 17 |
| Nemertea | 0 | 0 | 0 | 0 | 1 | 0 |
| Platyhelminthes | 2 | 2 | 2 | 0 | 6 | 6 |
| Porifera | 0 | 1 | 0 | 0 | 0 | 0 |
| Tunicata | 1 | 0 | 0 | 0 | 0 | 0 |
| Undetermined | 6 | 9 | 6 | 8 | 19 | 23 |
| Total | 46 | 50 | 37 | 31 | 57 | 71 |
