# Supplementary material for: Eukaryotic Richness in the Abyss: Insights from Pyrotag Sequencing
Source: PLoS One. 2011 Apr 4;6(4):e18169. doi: 10.1371/journal.pone.0018169 (PMC3070721; doi:10.1371/journal.pone.0018169)
Supplement: Table S2 — Taxonomic composition of OTUs assigned to Foraminifera. (DOC) [file pone.0018169.s003.doc]

Table S2.

Taxonomic composition of OTUs assigned to Foraminifera

| **Foraminifera** | **DSE1** | **DSE2** | **DSE3** | **DSE4** | **DSE5** | **DSE6** |
| --- | --- | --- | --- | --- | --- | --- |
| ENFOR1 | 2 | 0 | 0 | 1 | 4 | 2 |
| ENFOR3 | 4 | 4 | 9 | 2 | 7 | 5 |
| ENFOR4 | 1 | 1 | 1 | 0 | 1 | 5 |
| ENFOR5 | 3 | 1 | 1 | 2 | 1 | 1 |
| ENFOR7 | 0 | 1 | 0 | 0 | 0 | 1 |
| Globigerinacea | 1 | 0 | 1 | 0 | 1 | 1 |
| MON clade A | 0 | 1 | 1 | 0 | 0 | 0 |
| MON clade B | 0 | 1 | 0 | 0 | 0 | 1 |
| MON clade C | 0 | 1 | 1 | 0 | 0 | 0 |
| MON clade D | 2 | 0 | 1 | 0 | 0 | 1 |
| MON clade F | 0 | 1 | 0 | 0 | 0 | 0 |
| MON clade G | 0 | 0 | 1 | 0 | 0 | 1 |
| MON clade J | 0 | 0 | 0 | 0 | 0 | 2 |
| MON clade L | 1 | 0 | 0 | 1 | 0 | 2 |
| MON clade M | 5 | 5 | 2 | 2 | 1 | 0 |
| MON Vanhoeffenella | 3 | 6 | 1 | 0 | 0 | 2 |
| MON Bathysiphon | 0 | 0 | 0 | 0 | 1 | 0 |
| MON Tinogullmia | 0 | 0 | 0 | 0 | 1 | 0 |
| MON Conqueria | 0 | 0 | 0 | 2 | 0 | 3 |
| other monothalamids | 0 | 0 | 0 | 0 | 0 | 1 |
| Rotaliida | 2 | 2 | 1 | 2 | 5 | 7 |
| Textulariida | 7 | 6 | 1 | 2 | 1 | 5 |
| Undetermined | 4 | 7 | 2 | 2 | 3 | 9 |
|  | 35 | 37 | 23 | 16 | 26 | 49 |
